# Supplementary material for: Atomic-scale observation of nucleation- and growth-controlled deformation twinning in body-centered cubic nanocrystals
Source: Nat Commun. 2024 Jan 16;15:560. doi: 10.1038/s41467-024-44837-8 (PMC10791697; doi:10.1038/s41467-024-44837-8)
Supplement: Supplementary file 1 — Supplementary Information [file 41467_2024_44837_MOESM1_ESM.pdf]

Supplementary Information

Atomic-scale observation of nucleation- and growth-controlled deformation twinning in body-centered cubic nanocrystals

Li Zhong[1,2], Yin Zhang[3], Xiang Wang[1], Ting Zhu[3]*, Scott X. Mao[1]*

[1]Department of Mechanical Engineering and Materials Science, University of Pittsburgh, Pittsburgh, Pennsylvania 15261, USA

[2]SEU-FEI Nano-Pico Center, Key Laboratory of MEMS of Ministry of Education, Southeast University, Nanjing 210096, China

[3]Woodruff School of Mechanical Engineering, Georgia Institute of Technology, Atlanta, Georgia 30332, USA

*Correspondence to: ting.zhu@me.gatech.edu (TZ); sxm2@pitt.edu (SXM)

Supplementary Figures

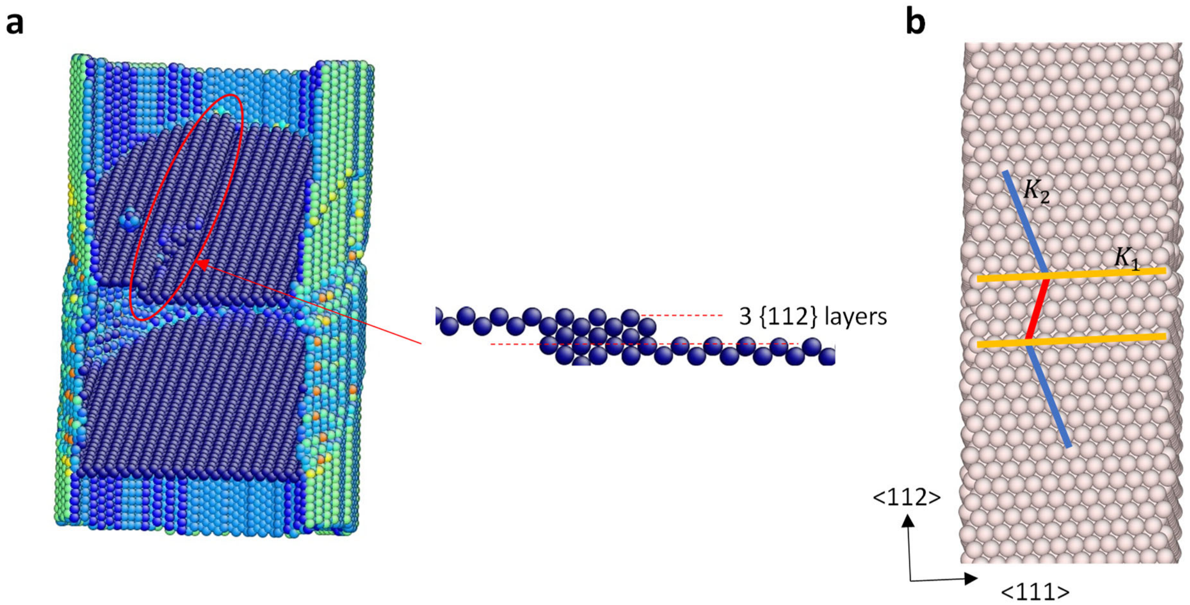

Supplementary Figure 1. MD results of twin structures in a <001>-oriented Ta nanowire with a diameter of 16 nm and a length of 40 nm. (a) Bunching of three atomic steps on successive (112) layers associated with three twin partials on a {112} CTB. (b) Atomic configuration of a deformation twin viewed from the <110> direction, showing the twinning element K_1 (twinning plane) and K_2 (conjugate twinning plane).

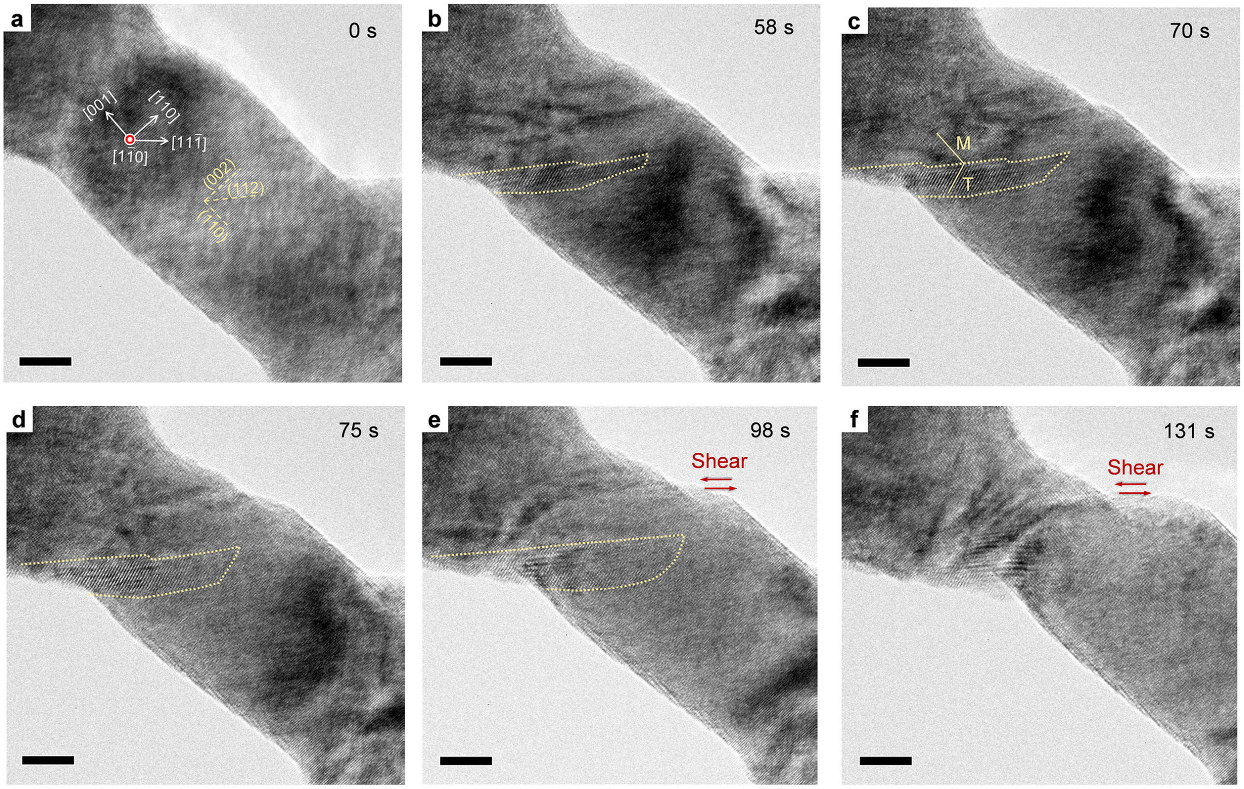

Supplementary Figure 2. Limited ductility due to a transition from twin thickening to dislocation-mediated deformation. a, Tensile loading of a [001]-oriented 18-nm-diameter Ta nanocrystal under a strain rate of ~1.5×10^{-3} s^{-1}. **b-d,** Slow twin growth featuring the formation of Moiré patterns and curved twin boundaries (outlined by yellow curves), consistent with the observations shown in Fig. 1. **e-f,** Transition from twin growth to dislocation-dominated plasticity as a result of accumulated elastic strain during twin thickening, leading to premature failure by localized shear. All scale bars are 5 nm.

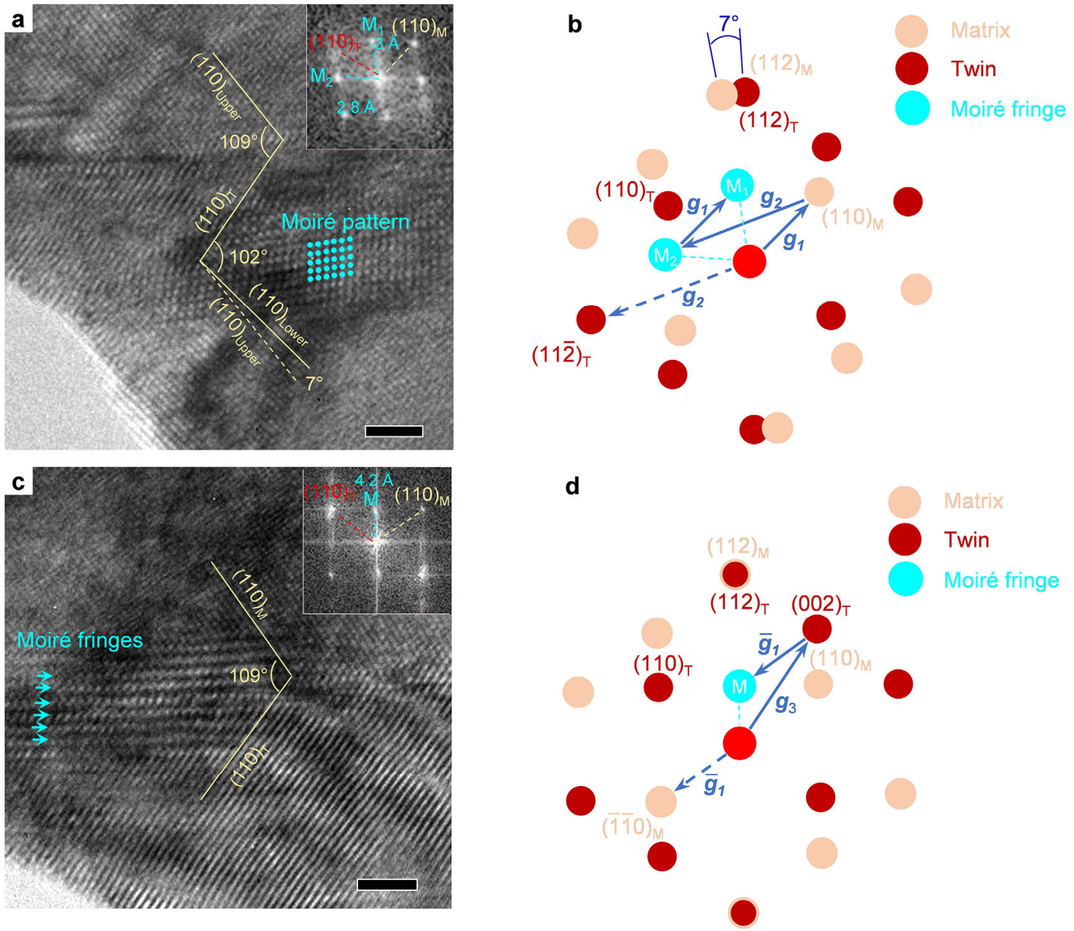

Supplementary Figure 3. Moiré fringes formed by overlapping of the projected lattice of parent and twinned crystals. a, High-resolution TEM image of the Moiré pattern formed during twin growth (see also Fig. 1f). Due to lattice distortion and bending during twin growth, there is a misorientation of 7° between the (110) planes in the twin (denoted as $(110)_T$) and the parent crystal below the twin boundary (denoted as $(110)_{Lower}$). The two diffraction spots associated with the Moiré pattern are indexed as M_1 and M_2 (inset in **a**), corresponding to a spacing of 3 Å and 2.8 Å, respectively, which is approximately two times the lattice spacing of the (112) planes. **b,** Replotting of diffraction patterns of both the parent crystal and the twin from the [1$\bar{1}$0] zone axis, showing that M_1 is formed by (110) reflection in the parent crystal ($\boldsymbol{g}_1$) followed by (11$\bar{2}$) reflection in the twin ($\boldsymbol{g}_2$), and M_2 is formed by further reflection by $\boldsymbol{g}_1$. **c,** Close-up view of the Moiré fringes at the upper twin boundary (see also Fig. 1i). The diffraction spots responsible for the Moiré fringes (indicated by cyan arrows) with a spacing of 4.2 Å are indexed as M (inset in **c**). **d,** Replotting of diffraction patterns of both the parent crystal and the twin from the [1$\bar{1}$0] zone axis, demonstrating that M is formed by (002) reflection in the twin ($\boldsymbol{g}_3$) followed by ($\bar{1}\bar{1}$0) reflection in the parent crystal ($-\boldsymbol{g}_1$). All scale bars are 2 nm.

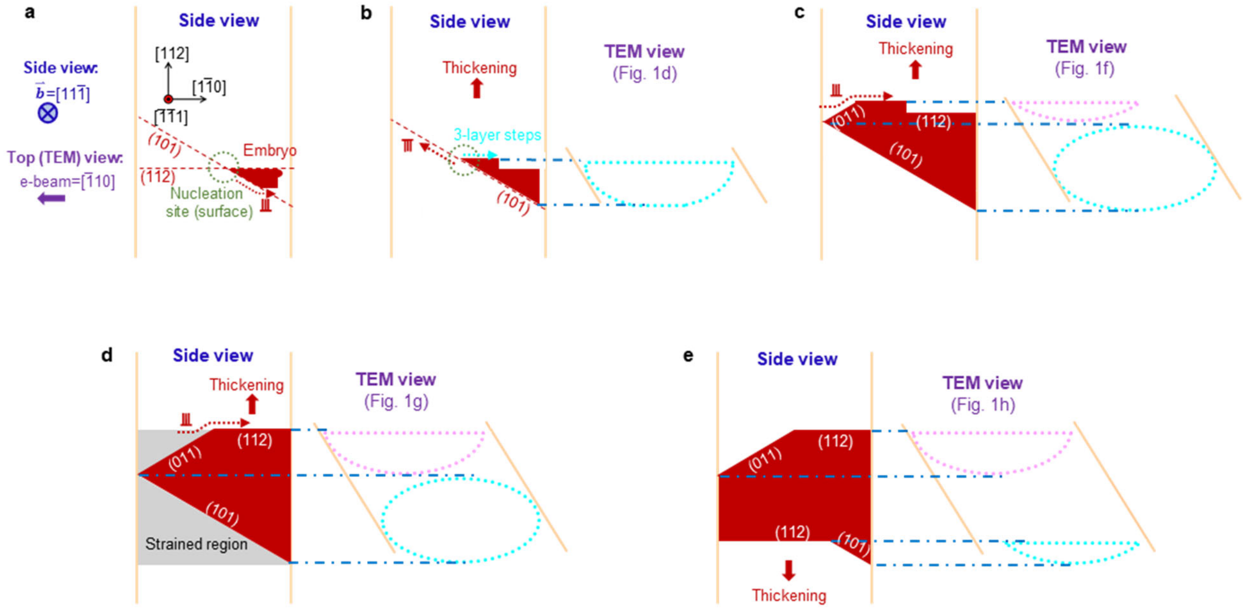

Supplementary Figure 4. Schematics of the 3D twin growth process based on *in situ* TEM observations shown in Fig. 1. To demonstrate the inclined twin boundaries, a series of sectional views (**a-e**) are provided with a viewing direction from the left side of the nanocrystal in Fig. 1 (i.e., parallel to the twinning shear direction of [11$\bar{1}$]). The twin embryo nucleated from the surface (circled in green) and grew into a wedge-shaped twin via a twin growth mechanism of cross-slip of screw dislocation, as shown in Fig. 4 (**a-b**). Due to the larger resolved shear stresses on the (101) and (011) slip planes compared to those on the ($\bar{1}$21) and (21$\bar{1}$) planes under [001] tensile loading, the full screw dislocations are more likely to cross-slip on (101) or (011) and then merge into the twin. Due to the uncertainty of cross-slip frequency and interaction site, the inclined twin boundaries are supposed to be on ($\bar{1}$21) and (21$\bar{1}$) with the lowest interface energy. The embedded twin tip then served as a site for subsequent upwards thickening (**b**). Due to the lack of CTB at the left side, 1/2[$\bar{1}\bar{1}$1] perfect screw dislocations were emitted towards the left, while groups of three 1/6[11$\bar{1}$] twinning partials were sent to the right and form three-layer steps on the CTB. The inclined ($\bar{1}$21) twin boundary may provide coherent matching at the interface, and thus could be kept stable during twin growth. After the twin tip has reached the left-side surface, further twin growth is governed by a cross-slip mechanism (**c-d**). To compare the proposed twin growth model to TEM observations, projections of the proposed inclined twin boundaries are viewed along the e-beam direction (TEM views in the right panel of **b-e**), which are consistent with the Moiré-fringed regions outlined in Fig. 1d,f,g,h, respectively.

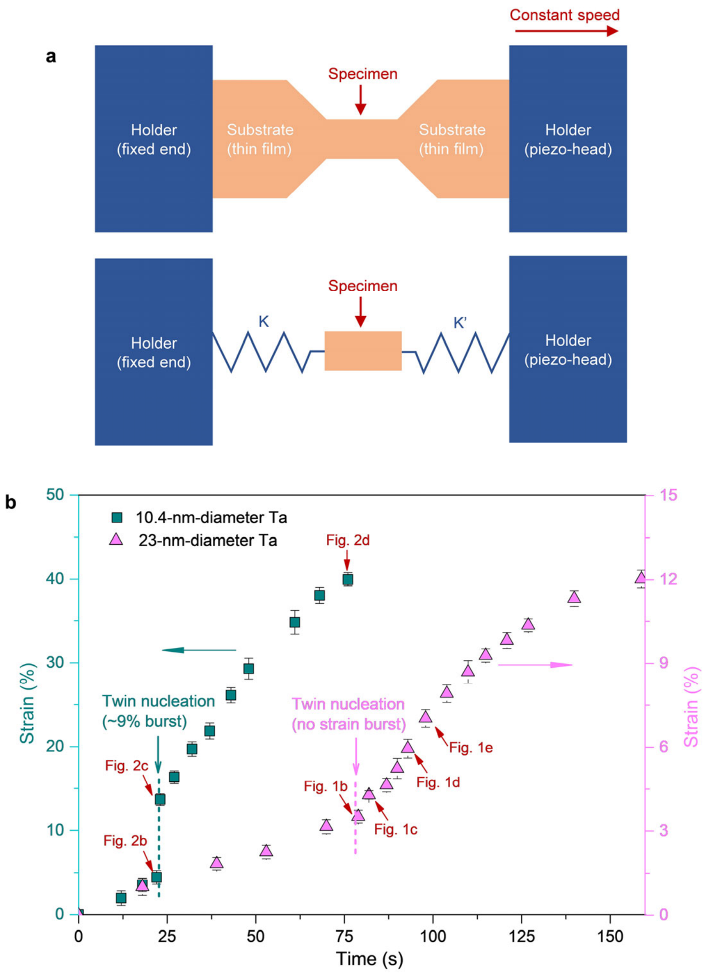

Supplementary Figure 5. Elongation during deformation twinning under <001> tension. a, Schematic illustration of tensile test setup (top) and the spring effect caused by the connecting thin film substrates (bottom). During tensile testing, the piezo-head is controlled to move at a near constant speed to apply tensile load to the specimen. In the meantime, the large thin-film substrates holding the specimen are also under straining and undergo elastic deformation, serving as springs. Due to the spring effect, a drop in the flow stress in the specimen leads to an increase in the specimen elongation rate (i.e., strain rate), and vice versa, even though the piezo-head is moving at a constant speed. As a result, a significant instantaneous drop in stress is accompanied by a strain burst in the strain evolution curve. **b,** Elongation against time for a 10.4-nm- (dark cyan curve; see also Fig. 2a-d) and a 23-nm-diamter Ta nanocrystal (magenta curve; 0-159 s of the deformation process in Fig. 1), respectively. A strain burst accompanying twin nucleation was observed in the former (~9%) but absent in the latter. Gradual reduction in the strain rate (i.e., reduction in slope) with subsequent twinning (the magenta curve) indicates increasing flow stress during twin growth in the 23-nm-diameter Ta nanocrystal. Error bars represent standard deviations in the estimated engineering strain from repeated measurements. Red arrows marked the data points collected from the corresponding images in Figs. 1 and 2. Source data are provided as a Source Data file.

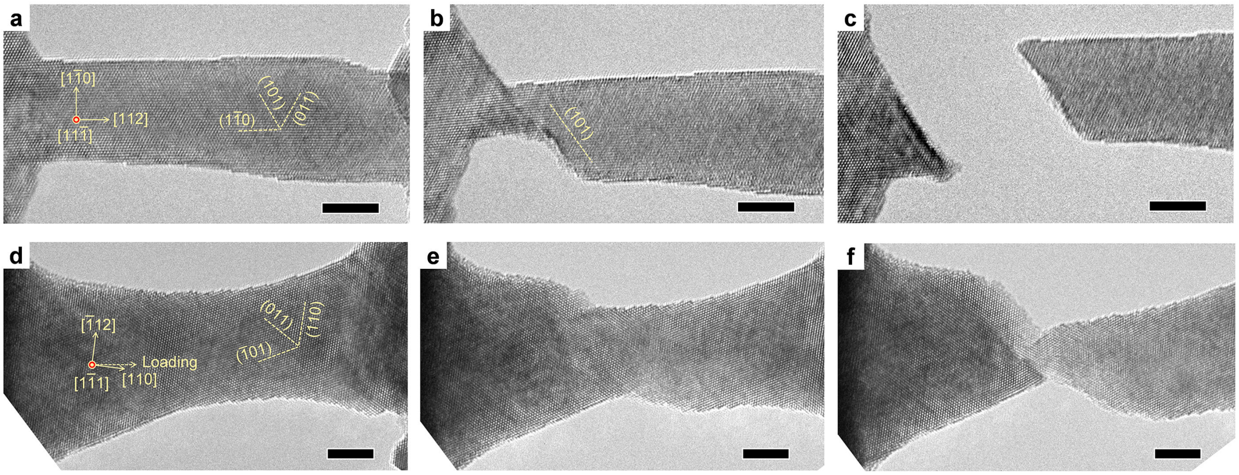

Supplementary Figure 6. Plastic instability by localized shear during dislocation-mediated deformation of two Ta nanocrystals. The tensile loading directions are <112> in **a-c** and approximately <110> in **d-e,** respectively. Both nanocrystals were viewed along the <111> zone axis. All scale bars are 5 nm.

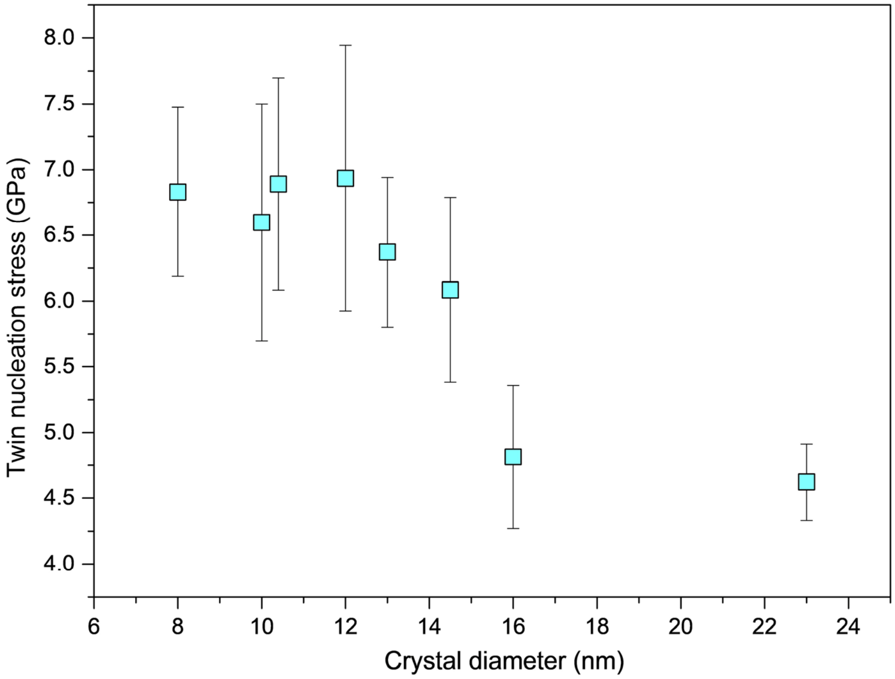

Supplementary Figure 7. Twin nucleation stress as a function of crystal diameter. Ta nanocrystals under <100> tension became yielded by twin nucleation, such that the yield stress was given by the corresponding twin nucleation stress. The nucleation stress was estimated based on the maximum overall elastic strain reached before yield. Error bars represent standard deviations in the estimated stresses from repeated measurements. Source data are provided as a Source Data file.

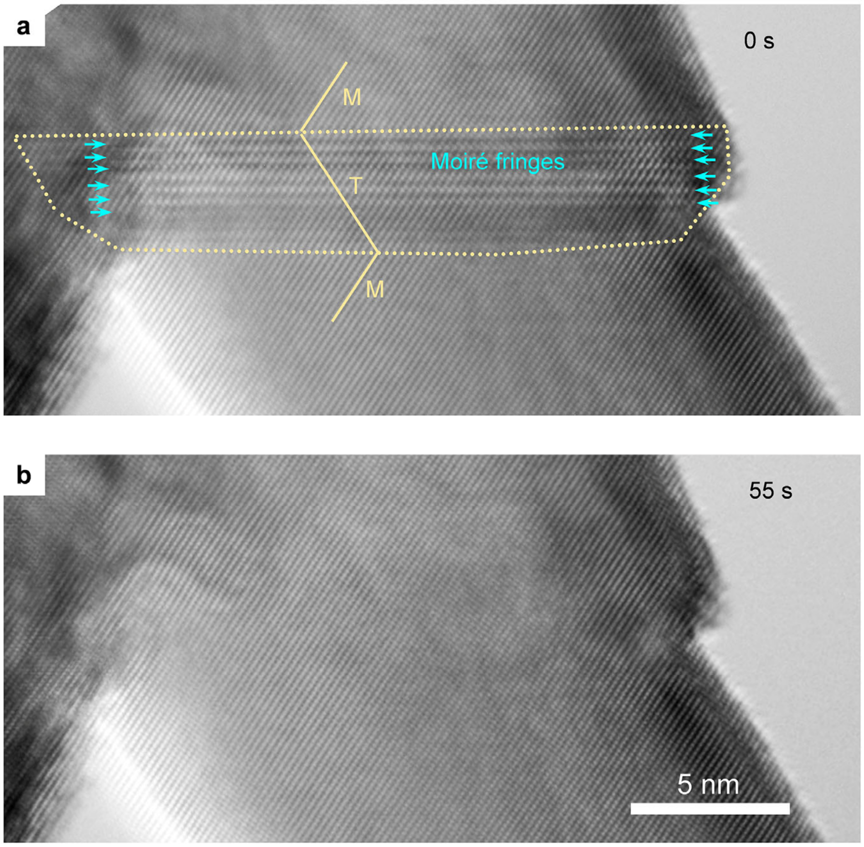

Supplementary Figure 8. Spontaneous detwinning in a W nanocrystal after unloading. a, A 4-nm-thick twin (outlined by yellow dotted lines) with Moiré fringes (denoted by cyan arrow heads) was formed under <110> compression. **b,** Detwinning proceeded rapidly and completed within 60 seconds after underloading.

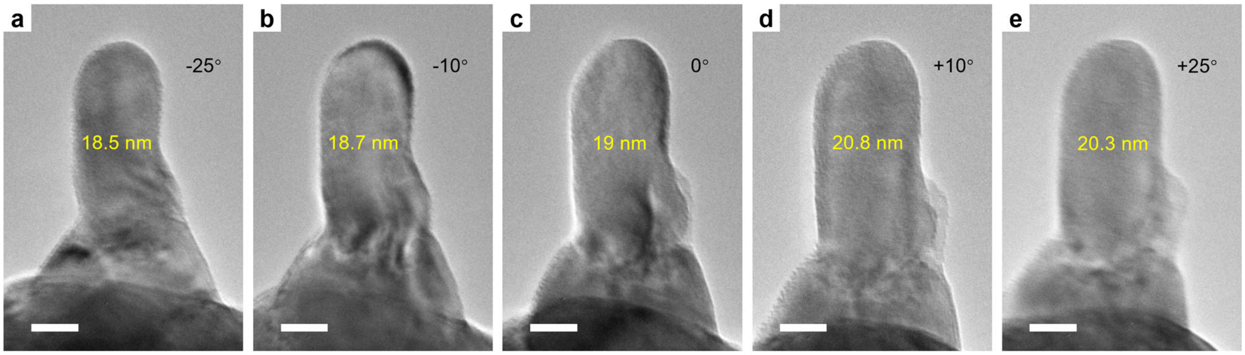

Supplementary Figure 9. Near-circular cross-section of a typical specimen prepared by the ultrafast liquid-quenching approach. a-e, Morphologies of the same specimen when it was rotated about its axial direction from -25° to 25° (i.e., a span of 50°). The maximum difference among diameters from different viewing directions is measured to be as small as ~10%. All scale bars are 10 nm.

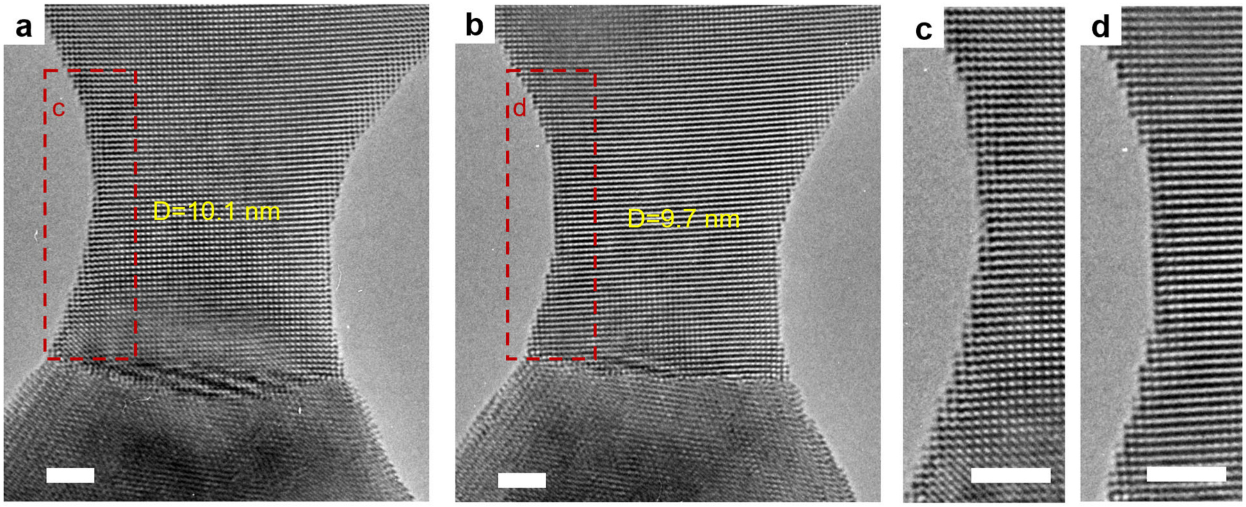

Supplementary Figure 10. Comparable geometry and surface defect characteristics between specimens in the current study. a-b, Two as-formed specimens with similar geometries. **c-d,** Enlarged views of regions boxed in red in (**a**) and (**b**). The surface defects of both samples are almost exclusively atomic steps, with a slight difference in distribution. All scale bars are 2 nm.
