# Supplementary material for: Atomic-scale observation of nucleation- and growth-controlled deformation twinning in body-centered cubic nanocrystals
Source: Nat Commun. 2024 Jan 16;15:560. doi: 10.1038/s41467-024-44837-8 (PMC10791697; doi:10.1038/s41467-024-44837-8)
Supplement: Supplementary file 3 — Description of Additional Supplementary Information [file 41467_2024_44837_MOESM3_ESM.pdf]

Description of Additional Supplementary Files

File Name: Supplementary Data 1
Description: Data for Figure 2h, Supplementary Figure 5b, and Supplementary Figure 7.

File Name: Supplementary Movie 1
Description: (see also Fig. 1a-h) Slow twin growth in a 23-nm-diameter Ta nanocrystal under <001> tension featured by slow lateral propagation of twinning partials and formation of inclined twin boundaries (evidenced by the formation of Moiré fringes). The movie is played at a speed of 15×.

File Name: Supplementary Movie 2
Description: (see also Fig. 2a-d) Facile twin thickening in a 10.4-nm-diamter Ta nanocrystal under [001] tension with a viewing direction of [1$\bar{1}$0]. Yielding (twin nucleation) was accompanied by a burst to the overall strain of ~14%, and followed by uniform elongation to ~40%. Because the twin plane is not in parallel with the [1$\bar{1}$0] viewing direction, an atomically resolved image of the twin is absent. However, the crystal reorientation (Fig. 2d-f) and the 40% maximum elongation (very close to the theoretical value of 41.4%) confirm deformation twinning in Movie S2. The movie is played at a speed of 6×.

File Name: Supplementary Movie 3
Description: (see also Fig. 2i-l) Rapid twin growth in a 12-nm-diamter Ta nanocrystal under [001] tension with a viewing direction of [100]. Plastic deformation initiated by a ~15% strain burst and continued by progressive elongation to a strain of ~37%. Two parallel interfaces were formed upon yield and migrated during subsequent deformation. These interfaces (twin boundaries) are parallel to the [021] direction, consistent with the projection of the twin plane. The movie is played at a speed of 6×.

File Name: Supplementary Movie 4
Description: Atomistic simulation results of twin nucleation and growth mediated by an array of screw dislocations in single-crystal Ta. The leading screw dislocation forms a three-layer twin embryo under an applied anti-plane shear stress. Then a follow-up screw dislocation catches up and merges into the twin embryo by cross-slip, resulting in a six-layer twin. Such a twin thickening process can repeat by the follow-up screw dislocations one by one, as shown in this Movie with two screw dislocations behind the three-layer twin embryo.

File Name: Supplementary Movie 5
Description: Atomistic simulation results of twin nucleation and growth mediated by an array of screw dislocations in single-crystal Ta. The leading screw dislocation forms a three-layer twin embryo under an applied anti-plane shear stress. Then a follow-up screw dislocation catches up and merges into the twin embryo by cross-slip, resulting in a six-layer twin. Such a twin thickening process can repeat by the follow-up screw dislocations one by one, as shown in this Movie with three screw dislocations behind the three-layer twin embryo.
